# Agreement between measured weight, height and BMI and Web-based self-reported data in participants of the 1993 Pelotas Birth Cohort, Brazil: a cross-sectional validation study

**DOI:** 10.1590/S2237-96222023000200013

**Published:** 2023-07-28

**Authors:** Vivian Hernandez Botelho, Aluísio J. D. Barros, Renata Gonçalves de Oliveira, Rafaela Costa Martins, Helen Gonçalves, Ana M. B. Menezes, Cauane Blumenberg

**Affiliations:** 1 Universidade Federal de Pelotas, Programa de Pós-Graduação em Epidemiologia, Pelotas, RS, Brazil; 2 Causale Consultoria, Pelotas, RS, Brazil

**Keywords:** Validation Study, Body Weights and Measures, Body Mass Index, Measurement of Correlation., Estudio de Validación, Pesos y Medidas Corporales, Índice de Masa Corporal, Medidas de Correlación, Estudo de Validação, Pesos e Medidas Corporais, Índice de Massa Corporal, Medidas de Correlação

## Abstract

**Objective:**

to evaluate the agreement between measured height, weight, and body mass index (BMI) during the 22-year follow-up of the 1993 Pelotas Birth Cohort, state of Rio Grande do Sul, Brazil, and self-reported data during the online follow-up of the *coortesnaweb*.

**Methods:**

this was a cross-sectional validation study; agreement was assessed by means of Lin’s concordance correlation coefficient for continuous measures and weighted Kappa for nutritional status; Spearman’s rank correlation coefficient was used to estimate the correlation between measurements.

**Results:**

a total of 783 participants were included; it could be seen high correlation and high agreement between the measured height (r = 0.966; ρ = 0.966), weight (r = 0.934; ρ = 0.928), and BMI (r = 0.903; ρ = 0.910) and Web-based self-reported data; there was no correlation between mean difference and the time interval between measurements.

**Conclusion:**

using the Internet to collect self-reported anthropometric measurements is as valid as the traditional method.

## Resumo

**Objetivo:**

avaliar a concordância entre altura, peso e índice de massa corporal (IMC) aferidos durante o acompanhamento dos 22 anos da coorte de nascimentos de Pelotas, Rio Grande do Sul, Brasil, de 1993, e dados autorrelatados durante o acompanhamento *online* da *coortesnaweb*.

**Métodos:**

estudo transversal de validação; a concordância foi avaliada pelos coeficientes de correlação de Lin para medidas contínuas e de Kappa ponderado para o estado nutricional; utilizou-se a correlação de Spearman para estimar a correlação entre as medidas.

**Resultados:**

783 participantes foram incluídos; observou-se alta correlação e alta concordância entre as medidas de altura (r = 0,966; ρ = 0,966), peso (r = 0,934; ρ = 0,928) e IMC (r = 0,903; ρ = 0,910) aferidas e as autorrelatadas via internet; não houve correlação entre as diferenças médias e o intervalo de tempo entre as medidas.

**Conclusão:**

utilizar a internet para coletar medidas antropométricas autorrelatadas é um método válido, comparado ao método tradicional.

## RESUMEN

**Objetivo:**

evaluar la concordancia entre la altura, el peso y el índice de masa corporal (IMC) medidos durante el acompañamiento de 22 años de la cohorte de nacimientos de Pelotas de 1993 y autoinformado durante el seguimiento en línea de la coortesnaweb.

**Métodos:**

estudio metodológico de validación. Se utilizó el coeficiente de correlación de Lin para medidas continuas, y de Kappa ponderado para el estado nutricional y la correlación de Spearman para la correlación entre medidas.

**Resultados:**

se incluyeron 783 participantes, con alta correlación y concordancia entre las medidas de talla (r = 0,966; ρ = 0,966), peso (r = 0,934; ρ = 0,928) e IMC (r = 0,903; ρ = 0,910) medidos y autoinformados vía web. No hubo correlación entre las diferencias de medidas y el intervalo de tiempo entre las mediciones.

**Conclusión:**

el uso de internet para recopilar variables antropométricas autoinformadas es válido en comparación con el método tradicional.

**Table:** 

Study contributions	
**Main results**	We observed high agreement and high correlation between measured height, weight and BMI during a face-to-face assessment and Web-based self-reported data. There was no difference in measurements between sexes and they were not correlated with the time interval between data collections.
**Implications for services**	The Internet can serve to collect anthropometric data, especially weight and height, optimizing the logistics of monitoring people who use health services, reducing costs and professional workload.
**Perspectives**	To continue advancing in this area, it is important that further studies integrate Web-based data collection to traditional methods. The use of the Internet can serve as a complementary method for monitoring health care users.

## INTRODUCTION

Face-to-face data collection is the most widely used method in epidemiologic studies. Despite the advancement of broadband internet access, its use for data collection by researchers has stood out.^1^ ^)-(^ ^3^ Web-based questionnaires allow collecting data quickly, more broadly, and at low cost;^4^ however, if designed without methodological rigor, these survey tools may have low response rates and limitations regarding the validity of the data obtained.^5^

Most Web-based epidemiologic studies are restricted to investigating issues that are less sensitive to self-report, replicating traditional paper-based questionnaires in an electronic version.^6^ ^),(^ ^4^ However, the number of Web-based studies collecting self-reported anthropometric data, especially height and weight, is increasingly used.^7^ Anthropometric data, particularly height and weight, are essential for monitoring public health issues in populations and their association with diseases and health conditions such as obesity.^7^ ^),(^ ^8^

Web-based anthropometric data collection can reduce logistical complexity, given that it is not necessary to train a team to measure these data, and there is no need to acquire or move equipment, in addition to reducing costs. These factors have motivated new Web-based studies. Furthermore, some studies have evaluated anthropometric measurements, comparing them with self-reported data using a face-to-face self-administered questionnaire, and thus, have demonstrated high agreement between both methods.^8^ ^)-(^ ^10^

However, there is no consensus on the validity of Web-based self-reported information, a situation in which there are not any team members to collect the data.^5^ This study seeks to fill this gap by contributing to the literature on data collection methods, i.e., to verify the validity of the Web-based self-reported measurements according to their comparison to those measured during a face-to-face assessment, which may help in further studies, as well as in the monitoring of these measurements by the Brazilian National Health System (*Sistema Único de Saúde* - SUS).

The objective of this study was, precisely, to evaluate the agreement between (i) measured height and weight and (ii) the body mass index (BMI) calculated during the 22-year follow-up of the 1993 Pelotas Birth Cohort, self-reported via the Internet during the online follow-up of the *coortesnaweb*.

## METHODS

This was a cross-sectional validation study, based on data from the 1993 Pelotas Birth Cohort, which, during perinatal follow-up, included 5,249 live-born infants in 1993 in the city of Pelotas, state of Rio Grande do Sul, Brazil. Data were obtained from two follow-ups of the birth cohort, one conducted face-to-face, and another via the internet. In the period of the face-to-face follow-up, 2015-2016, the participants were 22 years old and 3,810 of them answered the questionnaire and underwent a series of medical examinations. Anthropometric measurements of weight and height, and BMI were used in this study. Weight was measured using a scale (COSMED) with 10 g precision; and height was measured with stadiometer (Haspenden Portable Stadiometer) with 0.1 cm precision. BMI was calculated by dividing body weight by height squared (BMI = weight/height^2^).^11^ All height and weight measurements were performed by professionals trained according to its standardization. The anthropometric measurements, obtained in the clinical environment, underwent strict quality control every week to avoid any type of measurement bias.

In 2018, the members of the 1993 Birth Cohort who took part in the follow-up at 22 years of age granted the interview alone and stated that they had internet access (n = 3,537). Therefore, they received an invitation, sent via social media and e-mail, to take part in *coortesnaweb* - a Web-based platform, developed to collect multi-purpose epidemiological data, through Web-based self-administered questionnaires.^12^ Of the 3,537 individuals eligible to participate in the study, 1,306 registered on the platform. One of the questionnaires applied by *coortesnaweb* contained questions about body measurements, including weight and height - “How much do you weigh?” and “How tall are you?” -, at the time the questionnaire was compiled. The information from these two questions was used in the BMI calculation. The participants’ answers to the request to enter their weight and height values via the internet had their validation and consistency checked in real time: the questionnaire informed the participant of the expected number format and the range of acceptable values. Any inconsistency in the data entered onto the platform generated a warning to guide participants on how to enter valid values. After all, it is presumed that the absence of the researcher at the time of reporting weight and height via the internet tends to reduce the social acceptability bias of these measures.

Of the 1,306 members of the 1993 Birth Cohort registered on the platform, 783 had complete information on weight, height and BMI in both follow-ups, resulting in the analytic sample for the present study (Supplementary Material 1).

Weight and height values, measured in kilograms and centimeters, were analyzed continuously, as well as BMI, in kg/m² and classified into four categories: underweight (< 18.5 kg/m²), eutrophy (between 18.5 kg/m² and 24.9 kg/m²), overweight (between 25.0 kg/m² and 29.9 kg/m²) and obesity (> 30 kg/m²).^13^

Regarding data analysis, the sample was described in absolute and relative values, according to sex (male, female), race/skin color (White; Black; mixed race; Asian), schooling (in complete years of study) and asset index (in quintiles). The characteristics of the samples followed up at 22 years of age and who participated in the *coortesnaweb* were compared from the marginal homogeneity using the Stuart-Maxwell test.

The mean difference was estimated using Student’s t-test for paired samples. The difference between BMI categories was analyzed based on marginal homogeneity using the Stuart-Maxwell test. The agreement with the BMI category was evaluated by means of the weighted Kappa statistic, applying the formula for constructing the weighting matrix: 1−{(*i*−*j*)/(*k*−1)}^2^, where *i* and *j* represent the row and column indices of the matrix and the maximum number of categories of the variables. The agreement between continuous numerical variables was identified by means of Lin’s concordance correlation coefficient and the correlation using Spearman’s rank correlation coefficient, with confidence intervals estimated by means of 1,000 bootstrap repetitions.

The correlation between the difference in height, weight and BMI measurements and the time difference (in months) between both follow-ups was also estimated using Spearman’s rank correlation coefficient, visualized in the form of scatter plots. The difference between the mean weight and mean height of the participants according to sex was presented in Kernel density plots. Correlation and agreement according to schooling groups and asset quintiles were also estimated. For these analyses, the groups with the lowest level of schooling (in complete years of study: 0 to 4; 5 to 8) and the first three quintiles were grouped in order to obtain a sufficient sample size.

The analyses were performed using the Stata 17.0 (StataCorp LLC, College Station, TX, USA) and took into consideration a significance level of 5%. Correlation and agreement were considered as follows: absent (when the values were equal to 0.00); limited (values between 0.01 and 0.19); weak (between 0.20 and 0.39); moderate (from 0.40 to 0.59); strong (from 0.60 to 0.79); and high (when the values were equal to or greater than 0.80).^14^

The anthropometric measurements obtained in a clinical examination, taken by professionals trained in standardized procedures, were considered the gold standard.

The study project was approved by the Research Ethics Committee of the Medical School of the Universidade Federal de Pelotas (CEP/FAMED/UFPel) on November 15, 2017 (Opinion No. 2,382,790). All individuals who agreed to participate in the study signed an online Informed Consent Form, presented to them at the time of their registration on the *coortesnaweb* platform.

## RESULTS

**Figure 1:**
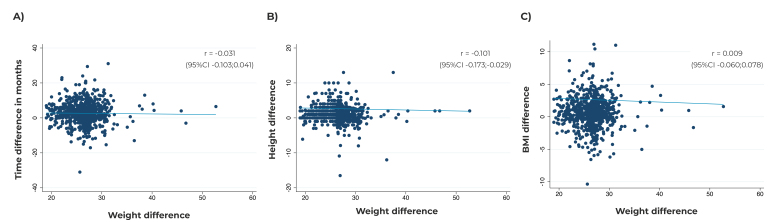
Correlation between the difference in weight (A), height (B) and BMI (C) and the time interval between the 22-year follow-up anthropometric measurements and *coortesnaweb*, Pelotas, state of Rio Grande do Sul, Brazil

Legend: BMI = body mass index; 95%CI = 95% confidence interval; r = Spearman’s rank correlation coefficient.

The sample of this study was comprised of 783 participants (60% of those registered), most of them female (65,8%), of White race/skin color (71,5%), with 12 years of schooling or more (49,9%) and belonging to the highest asset index quintile (the richest: 27,3%) (Table 1). Self-reported weight and height and the resulting BMI, recorded on the *coortesnaweb*, were, on average, 2.64 kg, 0.94 cm and 0.67 kg/m² higher compared to the same measurements taken face to face (Table 2). The time interval between the measurements and the differences between mean weight (r = -0.031), height (r = -0.101) and BMI (r = 0.009) were not correlated with each other (Figure 1).

**Table 1:** Characteristics of the sample according to sociodemographic variables of face-to-face follow-up at 22 and self-reported via *coortesnaweb* platform, Pelotas, state of Rio Grande do Sul, Brazil

Characteristics	Sample of the face-to-face follow-up of the 1993 Birth Cohort at 22 years old N (%)	**Analytical sample from *coortesnaweb*** **N (%)**	p-value
Sex			< 0.001
Female	2,027 (53.2)	515 (65.8)	
Male	1,783 (46.8)	268 (34.2)	
Race/skin color			< 0.001
White	2,262 (63.3)	539 (71.5)	
Black	538 (15.1)	87 (11.5)	
Mixed race	637 (17.8)	97 (12.9)	
Asian	137 (3.8)	31 (4.1)	
Schooling (in years of study)			< 0.001
0-4	111 (2.9)	2 (0.3)	
5-8	1,013 (26.6)	74 (9.5)	
9-11	1,560 (41.0)	316 (40.3)	
≥12	1,121 (29.5)	391 (49.9)	
Asset index (quintiles)			< 0.001
Q1 (the poorest)	761 (20.0)	87 (11.1)	
Q2	761 (20.0)	142 (18.2)	
Q3	761 (20.0)	158 (20.2)	
Q4	761 (20.0)	181 (23.2)	
Q5 (the richest)	760 (20.0)	213 (27.3)	

**Table 2:** Agreement and correlation between anthropometric measurements collected during a face-to-face assessment at 22 years old and self-reported via *coortesnaweb* (n = 783), Pelotas, state of Rio Grande do Sul, Brazil

Variables	22-year follow-up		*Coortesnaweb*		Agreement (95%CI^a^)	Correlation^b^ (95%CI^a^)
	N	% (95%CI^a^)	N	% (95%CI^a^)		
Sex						
Female	515	65.8 (62.4;69.0)	515	65.8 (62.4;69.0)	- ^c^	- ^c^
Male	268	34.2 (31.0;37.6)	268	34.2 (31.0;37.6)	- ^c^	- ^c^
Nutritional status					0.805^d^ (0.787;0.812)	0.804 (0.773;0.834)
Underweight	39	5.0 (3.7;6.7)	29	3.7 (2.6;5.3)		
Eutrophy	397	50.7 (47.2;54.2)	358	45.7 (42,3;49,2)		
Overweight	225	28.7 (25.7;32.0)	246	31.4 (28.3;34.8)		
Obesity	122	15.6 (13.2;18.3)	150	19.2 (16.5;22.1)		
	Mean (95%CI^a^)	SD^e^	Mean (95%CI^a^)	SD^e^	Agreement (95%CI^a^)	Correlation^b^ (95%CI^a^)
Age (years)	22.2 (22.1;22.2)	0.4	24.7 (24.6;24.7)	0.3	- ^c^	- ^c^
Weight (kg)	70.2 (69.0;71.4)	16.8	72.8 (71.6;74.0)	16.9	0.928^g^ (0.919;0.938)	0.934 (0.922;0.946)
Height (cm)	166.3 (165.7;167.0)	9.4	167.3 (166.6;167.9)	9.5	0.966^g^ (0.961;0.970)	0.966 (0.957;0.975)
BMI^f^ (kg/m^2^)	25.3 (24.9;25.7)	5.5	26.0 (25.6;26.4)	5.5	0.910^g^ (0.898;0.922)	0.903 (0.886;0.920)

a) 95%CI: 95% confidence interval; b) Spearman’s rank correlation coefficient; c) The measurements of agreement and correlation for sex and age are not shown, given that they do not present change between the two follow-ups; d) Weighted Kappa statistic; e) SD: standard deviation; f) BMI: body mass index; g) Lin’s concordance correlation coefficient.

However, the correlation and agreement between mean weight (r = 0.934 and ρ = 0.928 respectively), height (r = 0.966 and ρ = 0.966 respectively) and BMI (r = 0.903 and ρ = 0.910 respectively) were high. A similar result was observed for nutritional status, although a lower percentage of eutrophic individuals and higher percentage of obese individuals were found on the *coortesnaweb*, when compared to the findings of the face-to-face follow-up (Table 2). The accuracy of classification according to nutritional status was 97.1% between the two follow-ups. Analyses stratified by sex also showed high correlation and agreement between weight, height and BMI measurements (all coefficients above 0.800). The correlation/agreement of nutritional status was high among females and moderate among males: r = 0.756 and ρ = 0.761, respectively (Table 3).

**Table 3:** Analyses of agreement and correlation stratified by sex, according to anthropometric measurements during a face-to-face assessment at 22 years of age and self-reported via *coortesnaweb* (n = 783), Pelotas, Rio Grande do Sul, Brazil

Variables	Female		Male	
	Agreement (95%CI^a^)	Correlation (95%CI^a^)	Agreement (95%CI^a^)	Correlation (95%CI^a^)
Nutritional status	0.823 (0.811;0.834)	0.826 (0.791;0.861)	0.761 (0.726;0.800)	0.756 (0.698;0.814)
Weight (kg)	0.921 (0.909;0.934)	0.921 (0.904;0.937)	0.914 (0.895;0.933)	0.919 (0.893;0.945)
Height (cm)	0.936 (0.925;0.946)	0.930 (0.906;0.954)	0.935 (0.920;0.950)	0.940 (0.915;0.966)
BMI^b^ (kg/m^2^)	0.917 (0.904;0.931)	0.910 (0.891;0.928)	0.889 (0.864;0.914)	0.884 (0.847;0.921)

a) 95%CI: 95% confidence interval; b) BMI: body mass index.

**Figure 2:**
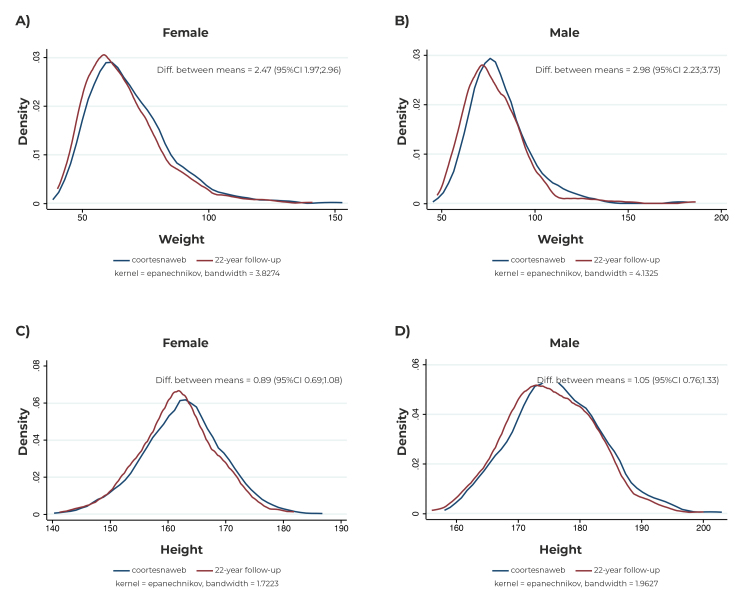
Distribution of measured weight (A, B) and height (C, D) according to the sex of the participants, in a face-to-face assessment at 22 years of age and self-reported via *coortesnaweb* (n = 783), Pelotas, state of Rio Grande do Sul, Brazil

Legend: Diff. between means = Difference between means; 95%CI= 95% confidence interval.

Differences between mean weight and height were also evaluated according to sex. Female reported a mean weight 2.47 kg (95%CI 1.97;2.96) higher on the *coortesnaweb* when compared to the face-to-face follow-up; for male, this difference was 2.98 kg (95%CI 2.23;3.73). Regarding height, female and male participants reported mean height 0.89 cm (95%CI 0.69;1.08) and 1.05 cm (95%CI 0.76;1.33) higher than that of the face-to-face follow-up, respectively. The differences between mean weight and height were similar between sexes (Figure 2).

The correlation and agreement between mean weight, height and BMI and the frequency of nutritional status were also evaluated according to schooling and asset index (Supplementary Materials 2 and 3). Regardless of the schooling group, the agreement between mean weight, height and BMI was high, higher than 0.900. With regard to nutritional status, the 0 to 8-year of schooling group showed the lowest agreement (ρ = 0.747), while the 9 to 11-year of schooling group reported the highest coefficient (ρ = 0.819). Similar results were observed when analyzed according to asset index quintiles.

## DISCUSSION

This study showed high correlation and agreement between Web-based height, weight and BMI, on the *coortesnaweb* platform, and face-to-face data collection, at 22 years of age of the 1993 Pelotas Birth Cohort. High correlations and agreement were observed regardless of sex, schooling and asset index of the individuals, indicating that Web-based self-reported anthropometric measurements is a valid form of data collection. High accuracy was also identified in the classification of nutritional status.

The findings of high correlation and agreement were similar to those of other related studies;^6^ ^),(^ ^15^ however, the main difference found was related to the time interval between the measurements: while the average time between the measured and self-reported data in this study was 26 months, in other studies, this interval ranged from three days to one month.^6^ ^),(^ ^15^ Thus, some variation related to weight was expected, given that the sample studied is in an age group in which changes may occur in routine, behaviors and food consumption.^16^ The same is not expected for height, whose change usually occurs in younger or older populations.^17^ After all, there was no correlation of the time interval between the measurements and the mean difference in height, weight and BMI, suggesting that this interval was not determinant of the differences observed.

The validity of anthropometric measurements self-reported by participants without the presence of a research team and those measured in a face-to-face interview has already been described in other studies. One of them, including young Australian adults whose average age was 24 years, found a correlation higher than 0.900 for measured height, weight and BMI and Web-based self-reported data - in line with what has been described here.^15^ Another study, conducted with adults whose average age was 38 years, also showed high agreement between measured weight and height and data self-reported by telephone.^18^

BMI category showed the lowest correlation and agreement, yet it had satisfactory estimated values (above 0.800 for both, correlation and agreement) and accuracy greater than 97% in the classification of nutritional status. BMI category is widely used in the literature as important information to describe the nutritional status of populations.^19^

The findings of this study did not identify differences between the measured data by sex of the participants, unlike what was described by Wilson et al.,^20^ whose research showed, in general, that females underestimated their weight, and that both sexes overestimated their heights, a result also pointed out in other studies, in which they self-reported their measurements with the presence of the research team.^9^ ^),(^ ^10^ The hypothesis of the authors of this report that there is no difference between sexes, may be related to the age group of the sample, composed of young adults, in addition to the fact that data was self-reported online and without the presence of the research team. The absence of an interviewer decreases the likelihood of concerns about social acceptance, in which information reported by participants may be biased by the desire to appear more acceptable to the research team.^21^

This study had some limitations, initially the eligibility for participation was restricted to individuals with internet access, which is why the sample is not representative of the 1993 Pelotas Birth Cohort. However, since the main objective of the study was to verify the validity of Web-based self-reported estimates, that was a necessary eligibility criterion. Nevertheless, sensitivity analyses of the agreement and correlation between the measurements, according to sex, schooling and asset index, demonstrated strong and high estimates. Another limitation of this study was the time interval between the Web-based self-reported anthropometric measurements and measured data; however, the correlation between the time interval and the mean difference was low. Other limitations of this study include the fact that participants with health problems and those who had attempted to lose/gain weight were not identified. However, the analyses showed high agreement for all measurements, demonstrating that the impact of such limitations on the results was limited.

This study has important advantages related to Web-based data collection, such as (i) responses are obtained more rapidly and (ii) real-time checking for possible typos or implausible values.^7^ The Web-based questionnaire used in this research included checks for weight and height data. Values with implausible formats, either due to errors in measurement or in the typing process, were flagged in order to alert the respondents. Unlikely values were also flagged to alert the respondents at the time of the questionnaire compilation, requesting that they be checked and corrected.

It can be concluded that the use of Web-based self-reported anthropometric measurements is a valid method that enables cost reduction, logistics optimization and speed up data collection among participants; in addition to being able to serve as a tool for monitoring the nutritional status of users of health services, both in the surveillance of nutritional status and in conducting translational research. The integration of this instrument with health information systems, such as ConecteSUS, can act as an integrated platform for monitoring users. Finally, it is suggested to investigate the agreement between other health data, which can be collected online and in a face-to-face interview, in order to verify their validity.

## Figures and Tables

**Supplementary Material 1:**
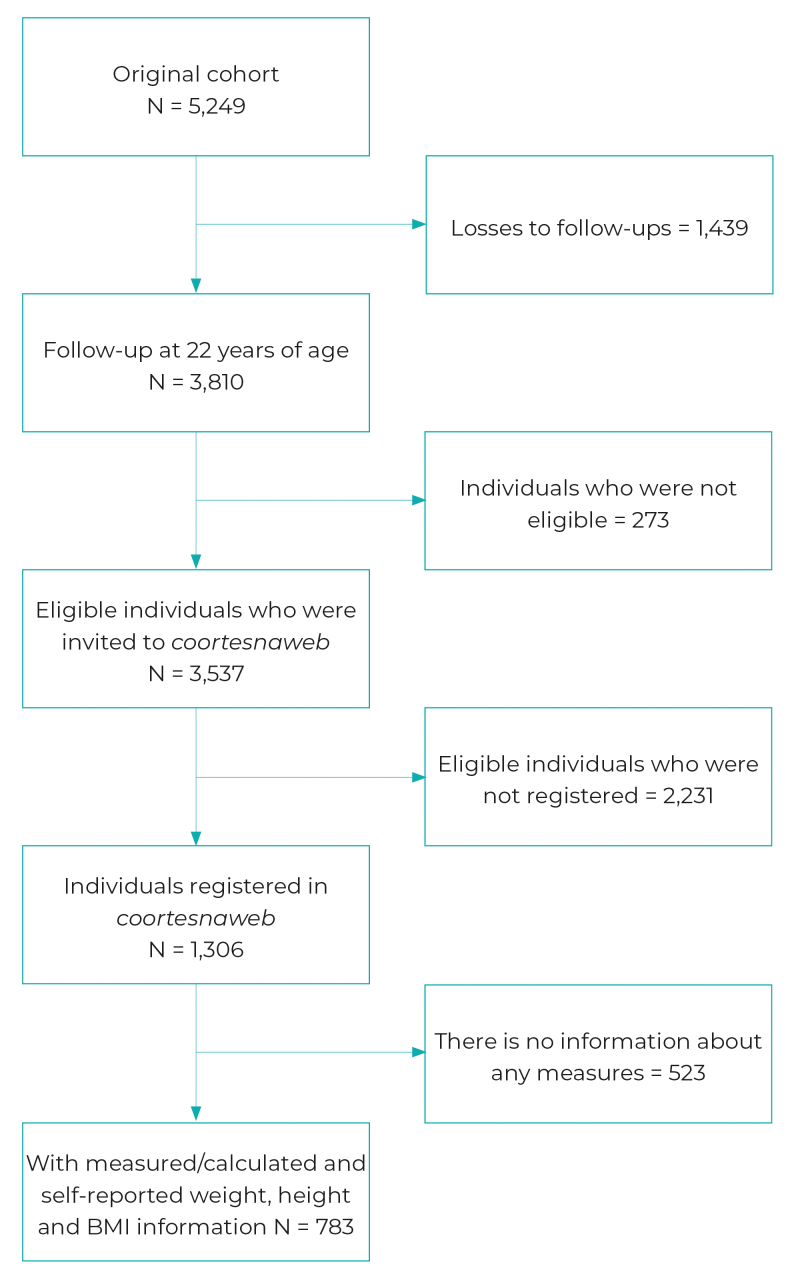
- Sampling flowchart for the 22-year follow-up and web *coortesnaweb*, Pelotas, state of Rio Grande do Sul, Brazil

## Supplementary Material 1

### - Sampling flowchart for the 22-year follow-up and web *coortesnaweb*, Pelotas, state of Rio Grande do Sul, Brazil

**Supplementary Material 2:** - Analyses of agreement and correlation stratified by schooling groups, according to anthropometric measurements during a face-to-face assessment at 22 years of age and self-reported via *coortesnaweb* (n = 783), Pelotas, state of Rio Grande do Sul, Brazil

Variables	0 to 8 years old		9 to 11 years old		12 years and older	
	Agreement (95%CI^a^)	Correlation (95%CI^a^)	Agreement (95%CI^a^)	Correlation (95%CI^a^)	Agreement (95%CI^a^)	Correlation (95%CI^a^)
Nutritional status	0.747 (0.653;0.848)	0.734 (0.595;0.873)	0.819 (0.785;0.834)	0.818 (0.773;0.862)	0.798 (0.772;0.814)	0.806 (0.762;0.850)
Weight (kg)	0.944 (0.920;0.968)	0.872 (0.794;0.950)	0.927 (0.912;0.943)	0.939 (0.923;0.957)	0.923 (0.908;0.937)	0.937 (0.922;0.952)
Height (cm)	0.935 (0.906;0.963)	0.927 (0.869;0.985)	0.961 (0.952;0.969)	0.963 (0.951;0.975)	0.976 (0.971;0.980)	0.977 (0.967;0.987)
BMI^b^ (kg/m^2^)	0.920 (0.886;0.955)	0.848 (0.756;0.940)	0.909 (0.890;0.928)	0.911 (0.884;0.938)	0.905 (0.887;0.923)	0.904 (0.882;0.927)

a) 95%CI: 95% confidence interval; b) BMI: body mass index.

**Supplementary Material 3:** - Analyses of agreement and correlation stratified by groups of asset index quintiles, according to anthropometric measurements during a face-to-face assessment at 22 years of age and self-reported via *coortesnaweb* (n = 783), Pelotas, state of Rio Grande do Sul, Brazil

Variables	Q1-Q3		Q4-Q5	
	Agreement (95%CI^a^)	Correlation (95%CI^a^)	Agreement (95%CI^a^)	Correlation (95%CI^a^)
Nutritional status	0.814 (0.797;0.821)	0.808 (0.765;0.851)	0.790 (0.760;0.824)	0.801 (0.760;0.842)
Weight (kg)	0.927 (0.914;0.941)	0.926 (0.906;0.946)	0.929 (0.915;0.942)	0.940 (0.926;0.954)
Height (cm)	0.950 (0.940;0.959)	0.948 (0.928;0.968)	0.976 (0.972;0.981)	0.978 (0.971;0.986)
BMI (kg/m^2^)	0.919 (0.903;0.934)	0.900 (0.872;0.927)	0.894 (0.874;0.914)	0.904 (0.880;0.927)

a) 95%CI: 95% confidence interval; b) BMI: body mass index.
